# Ecomorphological variability of *Arthrospira fusiformis* (Cyanoprokaryota) in African soda lakes

**DOI:** 10.1002/mbo3.125

**Published:** 2013-09-02

**Authors:** Mary Nakabungo Kaggwa, Alfred Burian, Steve Omondi Oduor, Michael Schagerl

**Affiliations:** 1 Department of Limnology, and Oceanography, University of ViennaAlthanstrasse 14, A-1190, Vienna, Austria; 2 Department of Ecology, Environment and Plant Sciences, Stockholm UniversityFrescati Backe, Svante Arrhenius V 21A, SE-106 91, Stockholm, Sweden; 3 Department of Biological Sciences, Egerton UniversityP.O Box 536, Egerton, Kenya

**Keywords:** *Arthrospira*, cell, environment, filament, phytoplankton morphology, saline lakes, *Spirulina*

## Abstract

The filamentous spirally coiled cyanoprokaryote *Arthrospira fusiformis* is found in extremely high densities in tropical soda lakes acting as driving force of the food web. We studied pronounced temporal morphological changes of *Arthrospira* in Kenyan soda lakes, Nakuru and Bogoria, and identified underlying key factors. Cell (diameter and height) and filament (height of coil, coil diameter, and number) dimensions were measured from weekly samples collected over a period of 16 months. In both lakes, medium-sized cells and large, widely coiled filaments prevailed most. Percentage of large, widely coiled filaments was promoted by elevated levels of soluble reactive phosphorus, wind speed, temperature and conductivity and the opposite for small filaments. Large, narrow-coiled filaments were associated with an increase in mainly *Arthrospira*-grazing zooplankton and cyanophage infections. Widely coiled spirals were promoted by increased turbulences. Based on fluorescence measurements, we found widely coiled filaments representing high vitality. From this study we were able to demonstrate for the first time morphological patterns of *Arthrospira* in nature. *Arthrospira* morphotypes are suitable for indicating the biological status in soda lakes as they are subjective and therefore reflective of what is happening in its habitat. Additionally, this outcome might be also of interest for commercial ′*Spirulina′* farms in enhancing high-quality production.

## Introduction

Effective resource exploitation under variable environmental conditions is one of the most important causes of intra- and interspecific morphological diversity in phytoplankton (Naselli-Flores and Barone 2000; Naselli-Flores et al. 2007). Available literature on morphological variability of phytoplankton indicates that environmental and biological constraints such as nutrients, light availability, and grazing pressure from herbivores influence phytoplankton morphology (Kagami and Urabe 2001; O'Farrell et al. 2007). It follows therefore that morphological traits adapted by phytoplankton are a reflection of changes and regularities of seasonal and/or environmental patterns. They not only show regular periodicities in weather patterns, but also reflect effects of perturbations or other disturbances in ecosystem (Naselli-Flores et al. 2007).

*Arthrospira fusiformis* (Voronichin) Komárek and Lund is a filamentous cyanobacterium that forms almost unialgal blooms in soda lakes of the East African Rift Valley (Vareschi 1978). These lakes are characterized by high levels of carbonate and bicarbonate contents and a pH of up to 11 (Vonshak 1997; Oduor and Schagerl 2007a). *A. fusiformis* is the main food source of the Lesser Flamingos, *Phoeniconaias minor* Saint-Hilaire (Vareschi and Vareschi 1984) linking *Arthrospira* abundance directly to the high number of these birds in African saline–alkaline lakes (Krienitz and Kotut 2010; Kaggwa et al. 2013). Lesser Flamingos are a big tourist attraction in Lakes Nakuru and Bogoria in Kenya, which has economic importance for local people (Harper et al. 2003; Schagerl and Oduor 2008; Krienitz and Kotut 2010). At times, the dominance of *A. fusiformis* suddenly crashes and the lake shifts toward an unstable pelagic community of different organisms, which cause high degrees of food insecurity for top-level consumers like fish and flamingos (Krienitz and Kotut 2010; Krienitz et al. 2013). *A. fusiformis* is commercially sold as “*Spirulina platensis*” for dietary supplement because of its high content of essential fatty acids, vitamins, proteins, and minerals (Jassby 1988; Tokuşogulu and Ünal 2003; Mühling et al. 2005; Zieliñska and Chojnacka 2009).

*Arthrospira fusiformis* strains have been observed to occur in a varied range of saline habitats which shows its ability to adapt to freshwater alkaline conditions as well as saline–alkaline and even hypersaline environments (Dadheech et al. 2010). In both natural and culture conditions, it shows high morphological variability (Mühling et al. 2003; Ballot et al. 2004; Wang and Zhao 2005). The main morphological feature of *A. fusiformis* is the patterned arrangement of its multicellular cylindrical trichome in an open helix. Trichomes are composed of cylindrical cells that undergo binary fission in a single plane, perpendicular to the main axis. Cell diameter ranges from about 3–12 μm, though occasionally it may reach up to 16 μm. The helix pitch typically ranges from 10–70 μm and its diameter from 20–100 μm. These two parameters which define the shape of the helix architecture are highly dependent on growth and environmental conditions (Vonshak and Tomaselli 2000).

Under laboratory conditions, Kebede (1997) detected differences in the length of its trichomes and degree of helicity when cultured at varying salinity levels expressing the physiological stress to which the cells were subjected to. The author observed that long trichomes occurred at the lowest salinity level (13 g L^−1^) while very short but closely coiled trichomes were dominating in Cl^−^ rich and highly saline media (55–68 g L^−1^). Additionally, very loose helices were distinctive for cultures grown in SO_4_^2−^ rich media. The helix feature in *A. fusiformis* shows high variability (Mühling et al. 2003; Wang and Zhao 2005) which probably is determined at the genetic level and induced by various environmental factors, hence the concept of ‘plasticity genes’ (Schlichting and Pigliucci 1993). This refers to the regulatory loci that directly respond to a specific environmental stimulus by triggering a specific series of morphogenic changes (Pigliucci 1996).

In the shallow African saline–alkaline lakes, it has already been observed that there are large temporal fluctuations in *A. fusiformis* biomass (Oduor and Schagerl 2007a; Schagerl and Oduor 2008; Krienitz and Kotut 2010). Even though such shifts in *A. fusiformis* biomass may go along with morphological changes, no comprehensive field study has been done on the morphological variability of *A. fusiformis* so far. In this study, we sought to address this gap by assessing the temporal morphological changes of *A. fusiformis* and identifying key environmental and biological variables that were responsible for these changes. Such shifts in morphology of the dominant primary producer probably have significant impacts on the food web structure, as grazing might be promoted or hindered by certain morphological features. Additionally, the study allowed evaluating the potential of *A. fusiformis* morphology as a reliable indicator of the biological stability in soda lakes.

## Material and Methods

### Study site

**Figure 1:**
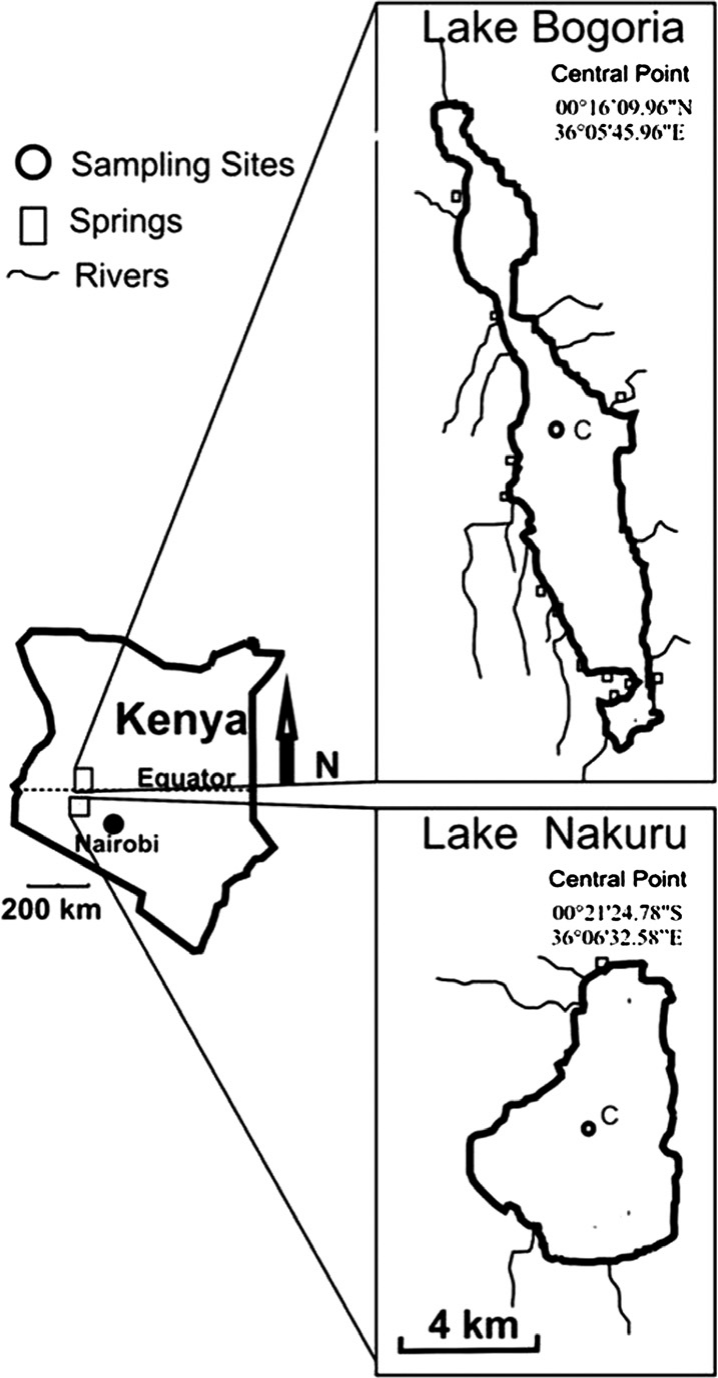
Map showing Kenya and the two studied saline–alkaline lakes, Nakuru and Bogoria.

This study was carried out in the two Kenyan Rift Valley lakes Nakuru and Bogoria (Fig. 1), which are known to host huge flocks of Lesser Flamingos with numbers sometimes rising over 2 million birds (Vareschi 1978), which is equivalent to 75% of its world population.

The sampling point in L. Nakuru is located in the central part at 00°21.387′S, 036°05.519′E which is representative of the whole ecosystem due to its polymictic character and the small size of the lake (Oduor and Schagerl 2007b). In L. Bogoria, the sampling point is located at 00°16.166′N, 036°05.766′E in the central region of the lake and is one of the deeper parts of the whole lake; it gives a good representation of the physical and chemical conditions when compared to the shallower northern and southern parts of the lake.

### Sample collection

Weather stations with complete data loggers of the HOBO RG3-M were installed at the shores of both lakes (Onset Computer Corporation, USA). Data were logged for wind speed, solar radiation, air temperature, and precipitation. All limnological parameters were sampled weekly from July 2008 to October 2009 (*n* = 130 sampling occasions). Measurements of environmental in situ parameters included light attenuation (PAR, Skye instruments, UK), pH, electrical conductivity, and water temperature (multiprobe WTW Multi 340i, Wissenschaftlich Technische Werkstätten Weilheim, Germany).

### Nutrient analysis

Filtered lake water was analyzed for NO_3_-N and SRP, which were both determined according to modified standard procedures (American Public Health Association, 1995) to cater for the high buffering capacity of the alkaline water.

### Cyanophages

Samples collected with a plankton net (30 μm mesh size) were fixed with glutaraldehyde to a final concentration of 2%. *A. fusiformis* filaments were prepared for transmission electron microscopy (Peduzzi P., Gruber M., Gruber M., Schagerl M., unpublished) to identify infected cells.

### Zooplankton

Surface water samples were taken with a 10 L Schindler sampler. Rotifers were concentrated with a 50 μm sieve, fixed with formalin (5% final concentration), and counted following the Utermöhl (1958) protocol. Presence of crustacean zooplankton was checked regularly with a plankton net (200 μm), but densities were constantly below 0.1 ind. L^−1^ and quantitatively not important. For ciliates, 250 mL of lake water was fixed with Bouin's solution (5%), stained using the Quantitative Protargol Staining Technique (QPS) by Montagnes and Lynn (1993), and counted with a compound microscope (1000x). For this study, we used only the biomass of *A. fusiformis* ingesting taxa based on feeding experiments with dominant rotifers and ciliates of African soda lakes (Burian et al. 2013).

### Phytoplankton

Lake water samples for microscopic examination, identification, and measurement of phytoplankton cells were taken 5 cm below the water surface at the sampling locations and the samples fixed with 5% formaldehyde. The taxa were identified with the aid of established identification keys (Kociolek and Spaulding 2003; Komárek 2003; Komárek et al. 2003; Shubert 2003). The cells of each taxon were enumerated and dimensions measured using an inverted microscope (Nikon Diaphot, Nikon, Tokyo) using 100× and 200× magnifications for *Anabaenopsis* and 400× for other taxa according to Utermöhl (1958; details for *A. fusiformis* see below). To estimate the biovolume of the various community taxa, we used geometric formulae of the shapes similar to the respective phytoplankton cells (Hillebrand et al. 1999). At least 30 cells for each identified taxon were measured to give the average size and biovolume. For conversion of cell volume (mm^3^) into biomass (mg), a conversion factor of 1 was used (Wetzel and Likens 1991).

### Morphological measurements of *A. fusiformis*

**Figure 2:**
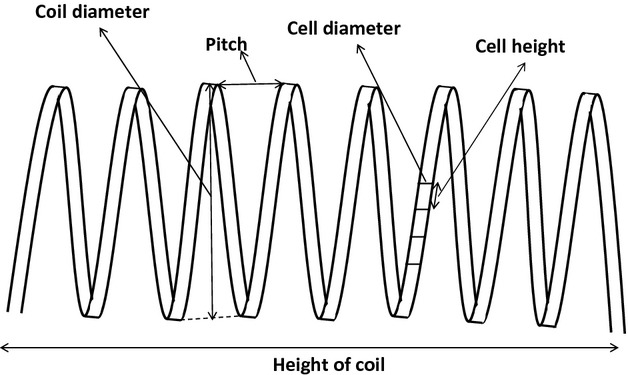
Illustration of cell and filament morphological dimensions of *Arthrospira*. Pitch = height of coil divided by number of full turns.

Cell diameter and cell height were measured from the same cell at a high resolution (1000×) using a Zeiss AXIO Imager M1 (Göttingen, Germany) microscope. Filament dimensions which included height of coil, coil diameter, and number of coils were measured from the same filament using an inverted microscope (Nikon Diaphot, Nikon, Tokyo) at 400× magnification (Fig. 2). We defined a surrogate parameter, the pitch of a filament, which is an indication of how tightly a filament is coiled. The pitch was calculated by dividing the height of a coil (μm) by the number of full turns. Each morphological variable was measured on 50 filaments per sample, which resulted in over 6000 measurements for each variable.

### Pulse amplified modulation (PAM) measurements

Pulse amplified modulation (PAM) fluorescence was used to estimate the overall photosynthetic performance of the phytoplankton community between June 2008 and May 2009. A raw sample was filtered (Whatman GF/C) at the lake shore under dim light and the filter clamped in a leaf clip. The clip together with the filter was then placed on a wet sponge in a black petri dish for 10 min in order to achieve dark acclimation (full relaxation of the photosystems). With a PAM fluorometer (FMS2, Hansatech, Great Britain), the initial (minimum) fluorescence (*F*_0_) was measured and after application of a saturating light impulse, the maximal fluorescence (*F*_m_) was estimated (intensity of saturation light pulse 85 relative units; duration of light pulse 0.7s). The so-called dark fluorescence yield *F*_*v*_/*F*_*m*_ or maximal operation efficiency of PSII was calculated as follows: variable fluorescence (*F*_*v*_) was obtained by subtracting *F*_0_ from *F*_*m*_. *F*_*v*_/*F*_*m*_ is a parameter that provides information about the physiological state of the photosynthetic organism (Baker 2008).

### Statistical analysis

**Figure 3:**
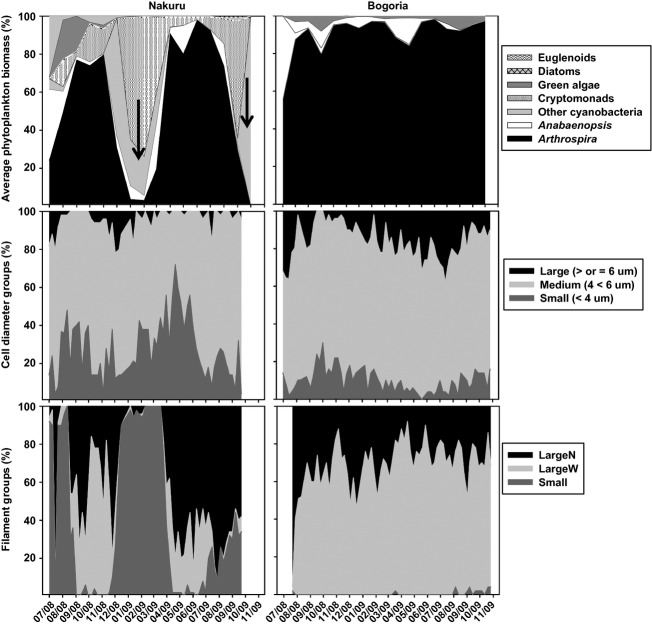
Temporal trends of average biomass of phytoplankton (top), cell diameter (middle), and filament (bottom) groups in L. Nakuru and L. Bogoria (arrow = period of *Arthrospira* biomass crash).

Data of both lakes were pooled for groupings and ordinations. Three groups of cell sizes (small: <4; medium: 4–6; large: >6 μm in diameter) were defined after examination of the size distribution of the cell diameter (Fig. 3). For pruning filament groups, a cluster analysis was run with SPSS 16.0 software: Standardized data for coil diameter (μm), number of coils per filament, and pitch (μm) were used as variables defining dissimilarity by applying Ward's method. Three filament groups were obtained (small, large + wide pitch = largeW, large + narrow pitch = largeN filaments). To test for homogeneity within the morphology groups, a multiresponse permutation procedure (mrpp) was performed with PC-ORD 5.33 software; applied distance measure was Sorensen (Bray-Curtis). Spearman's rank-order correlation was done to assess the relationship between the cell/filament morphology groups/*F*_*v*_/*F*_*m*_ and *A. fusiformis* biomass. Unconstrained ordination was used to explore the placements of cell and filament group distributions along synthetic axes (software package CANOCO version 4.5; Microcomputer Power, New York). Standard deviation of the gradients was <2.5, therefore the linear method of principal component analysis (PCA) was applied (Lepš and Šmilauer 2005). Samples were centered; supplementary environmental and biological variables were standardized and projected post hoc into the plots to assist interpretation of the group orientation. Variance inflation factors (VIF) for each variable were checked and variables with a VIF above 5 were excluded to minimize the problem of multicollinearity.

## Results

### Phytoplankton biomass

The main phytoplankton groups that occurred in lakes Nakuru and Bogoria are presented in Fig. 3. Most abundant were Cyanobacteria including *A. fusiformis*, *Anabaenopsis* spp., *Synechococcus minutus* West, *Synechocystis* sp., *Raphidiopsis* sp., and *Haloleptolyngbya alcalis* Dadheech, Mahmoud, Kotut et Krienitz. Other groups comprised cryptomonads, green algae (*Ankistrodesmus* sp., *Crucigenia* sp., and *Monoraphidium minutum* (Nägeli) Kormárková-Legnerová), and diatoms (*Nitzschia* sp. and *Navicula* sp.).

*Arthrospira fusiformis* contributed mostly to the overall biomass, though its abundance was highly variable in L. Nakuru compared to L. Bogoria (Fig. 3). In L. Nakuru, *Arthrospira* peaks were recorded from September to November, 2008 and from April to July 2009. *A. fusiformis* biomass crashed twice during the study period, between December 2008 to March 2009 and also from September to October 2009. For L. Bogoria, *A. fusiformis* dominated throughout the whole sampling period with no crashes observed (Fig. 3).

### Cell dimensions

**Figure 4:**
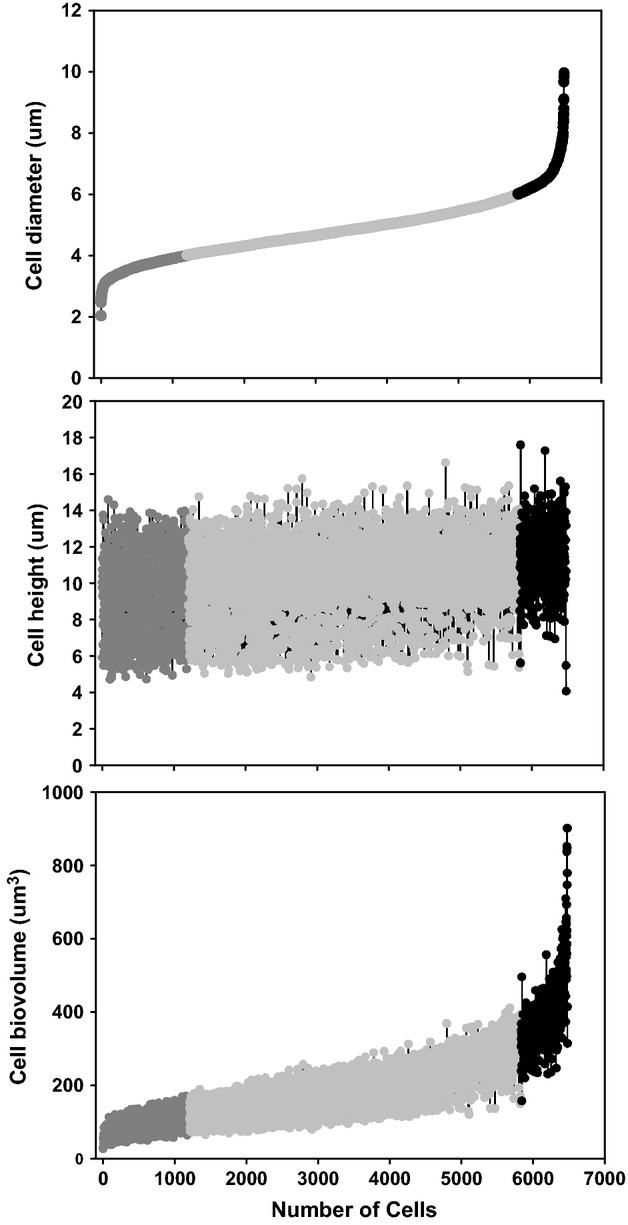
An overview of the different cell diameter size classes and it's relation with cell height and cell biovolume derived from combined data of both lakes (total *n* = 6469).

Cell size categories were based on diameter changes as illustrated in Fig. 4. A similar pattern was observed for cell diameter and biovolume. The pattern for cell height was comparable to that of the diameter and biovolume though it was obscured by considerable scattering. Multiresponse permutation procedure revealed that the groups were homogenous and well separated from each other (chance-corrected within-group agreement A = 0.46; *P* < 0.001). In both lakes Nakuru and Bogoria, cell diameter ranged from 2 to 9 μm with much more temporal variation in L. Nakuru compared to L. Bogoria (Fig. 3). In L. Nakuru, increased number of small cells was recognized from July to October 2008 and February to May 2009. Most of the cells in this lake were medium-sized category (68.1 ± 1.7%, SE), followed by small cells (30 ± 2%, SE), and a few large cells (4 ± 0.7%, SE). Spearman's rank-order correlation indicated that there was no significant correlation between the small (*P* = 0.65), medium (*P* = 0.58) as well as large (*P* = 0.09) cells and *A. fusiformis* biomass.

In L. Bogoria, a different scenario was depicted with no distinct changes in cell size groups observed over time. The dominant group was, however, the medium cells (74.6 ± 0.9%, SE) followed by the large cells (15.6 ± 1.2%, SE) and fewer small cells (9.8 ± 0.8%, SE). There was also no significant correlation observed between the small (*P* = 0.96), medium (*P* = 0.93) as well as large (*P* = 0.634) cells and *A. fusiformis* biomass.

**Figure 5:**
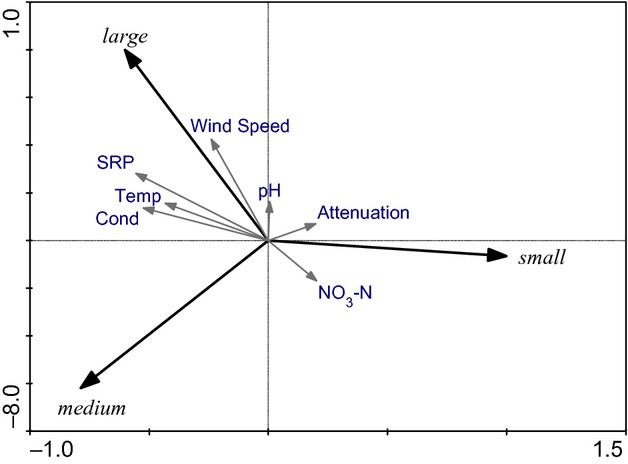
PCA-biplot based on cell groups (small, medium, large) and supplementary environmental variables.

The PCA-model revealed that about 42% of the differences in the cell groups could be explained by the post hoc projected environmental variables (Tables 1 and 2). The PCA biplot (Fig. 5) did not provide a clear distinction between the large and medium cells but a discrete separation was observed of large/medium cells from small cells along PC1. The occurrence of large cells was supported by elevated levels in SRP (soluble reactive phosphorous) concentration, wind speed, temperature, and conductivity whereas the opposite encouraged the prevalence of small cells. NO_3_-N, pH, and light attenuation played a minimum role in the cell group pattern.

**Table 1:** Summary of statistics of principal component analysis

	1	2	Total variance
Cell morphology axes			
Morphology–environment correlations	0.679	0.550	
Cumulative percentage variance			
of morphology data:	74.8	100.0	
of morphology–environment relations:	81.9	100.0	
Sum of all eigenvalues			1.000
Sum of all canonical eigenvalues			0.421
Filament morphology axes			
Morphology–environment correlations	0.773	0.703	
Cumulative percentage variance			
of morphology data:	70.5	100.0	
of morphology–environment relations:	74.4	100.0	
Sum of all eigenvalues			1.000
Sum of all canonical eigenvalues			0.568

**Table 2:** Environmental and biological variables (mean ± SD) of L. Nakuru and L. Bogoria during the study period

Variable	L. Nakuru	L. Bogoria
Soluble reactive phosphorus (mg L^−1^)	0.9 ± 1.1	3.1 ± 0.7
Water temperature (°C)	25.1 ± 1.9	28.6 ± 1.5
pH	10.1 ± 0.2	10 ± 0.2
Coefficient of attenuation	9.5 ± 2.9	8.2 ± 2.5
Specific conductivity (mS cm^−1^)	44.5 ± 18.2	67.2 ± 5.8
*Arthrospira* biomass (mg L^−1^)	39.1 ± 47.1	68.3 ± 29
*Arthrospira*-grazing zooplankton (mg C L^−1^)	2.4 ± 2.4	0.8 ± 1.4
Nitrate-N (mg L^−1^)	16.1 ± 9.5	11.3 ± 3.4
Wind Speed (km h^−1^)	4.2 ± 1.8	6.6 ± 1.7
Cyanophages (% infected cells)	1.0 ± 3.6	1.1 ± 3.6

### Filament dimensions

Three filament morphology groups were obtained from the cluster analysis (Table 3). Mrpp revealed a high homogeneity within groups and a significant separation (chance-corrected within-group agreement A = 0.43; *P* < 0.001). Similar to the cell group scenario, we observed high temporal dynamics in filament group shifts in L. Nakuru compared to L. Bogoria (see Fig. 3). In L. Nakuru, the abundance of small filaments peaked to 100% in July and August 2008 and for a more extended period from December 2008 to March 2009. Between September and November 2008, alternating dominance between largeW and largeN filaments was observed while from May till end of the sampling period, largeN filaments prevailed. The peaks of the small filaments appeared to coincide with those of small cells and *A. fusiformis* biovolume but there was no overall significant relationship between the small cells and small filaments (*P* = 0.76). On the other hand, the small filaments had a strong negative correlation with *A. fusiformis* biomass (*P* < 0.001, *r* = −0.75) while the largeW and largeN filaments were positively correlated to *A. fusiformis* biomass (*P* < 0.001, *r* = 0.69 and 0.45, respectively).

**Table 3:** Characteristics of filament morphology groups of *Arthrospira* obtained from cluster analysis for both lakes Nakuru and Bogoria (dimensions, mean ± SE; n total = 6469; Large-W = Large wide pitch filaments; Large-N = Large narrow pitch filaments)

Group	Sketch	*n*	Coil diameter (μm)	Coils/filament	Pitch (μm)
Small	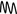	1336	36.6 ± 0.3	2.6 ± 0.1	11.1 ± 0.3
LargeW	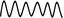	2848	50.1 ± 0.2	7.3 ± 0.1	63.2 ± 0.3
LargeN	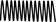	2285	60.5 ± 0.4	8.6 ± 0.1	12.6 ± 0.1

In L. Bogoria, the largeW filaments predominated throughout the study period at approximately 80% while the largeN filaments fluctuated around 20%. There was no significant relationship observed between small, largeW, and largeN filaments (*P* = 0.13, 0.60 and 0.81, respectively) and *A. fusiformis* biomass.

**Figure 6:**
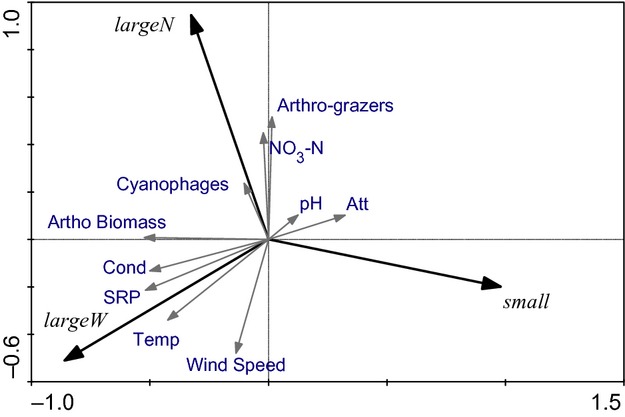
PCA-biplot based on filament groups and supplementary environmental variables *largeN* = largeN filaments; *largeW* = largeW filaments; *small* = small filaments.

Principal component analysis (PCA) for filament morphology indicated that about 57% of the differences in the filament groups (see Table 1 and 2) could be explained by post hoc projected variables. As was observed with the cell groups, there was a discrete separation of the small filaments from the largeN and largeW filaments along PC1 (Fig. 6). LargeW filaments dominated with increasing levels of conductivity, SRP, temperature, and wind speed. The occurrence of large N filaments was associated with increase in the *A. fusiformis*-grazing zooplankton (rotifers – *Brachionus plicatilis* Mueller and *Hexathra jenkinae* De Beauchamps and all ciliates larger than 60 μm), NO_3_-N, and cyanophage visibly infected cells. pH and light attenuation appeared to have played a negligible role in the group separations. As expected, *A. fusiformis* biovolume increased when the largeW and largeN filaments predominated. Small filaments were negatively coinciding with conductivity, SRP, temperature, and wind speed.

### PAM fluorescence measurements

**Figure 7:**
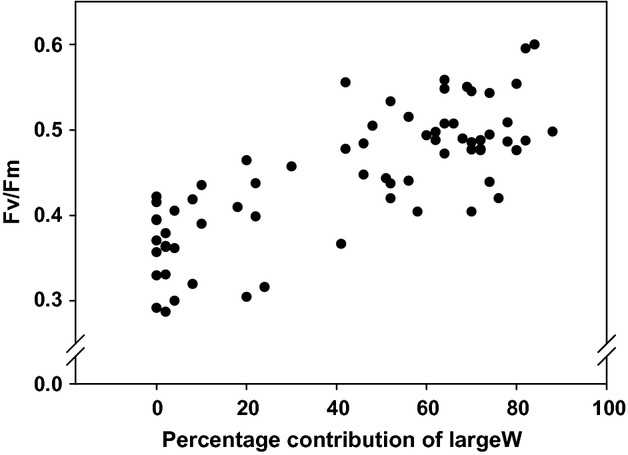
LargeW filament group related to *F*_v_/*F*_*m*_ values.

*F*_*v*_/*F*_*m*_ ranged between 0.20 and 0.68 in both lakes. Between largeW occurrence and *F*_*v*_/*F*_*m*_, a significant positive correlation was calculated (*r* = 0.703, *P* < 0.001, *n* = 69; Fig. 7). Also for largeN, a significant, although weaker positive relationship could be shown (*r* = 0.252, *P* = 0.037, *n* = 69). For small filaments, a negative correlation was obtained (*r* = −0.681, *P* < 0.001, *n* = 69).

## Discussion

The diverse ecological strategies adopted by phytoplankton can be related to their morphological variability (Reynolds 1997); this acclimation value is directed toward adapting the best fitting trait to the prevailing environmental template (Naselli-Flores et al. 2007). Morphological modification of *A. fusiformis* has been well studied under laboratory conditions such as variable irradiance (Wu et al. 2005; Helbling et al. 2006), temperature (Gao et al. 2008; Vonshak and Novoplansky 2008), and salinity (Kebede 1997). So far, no extensive field studies exist that indicate whether these changes that have been observed under extreme laboratory situations also occur in nature.

### Cell dimensions

The cell diameter measurements were in the range given by Vonshak and Tomaselli (2000). Even though cell height is not suitable for cell group classification as it is highly subjective to elongation during cell division as shown by the scatter observed in our measurements (Fig. 4), there was, however, a noticeable trend of larger diameter cells depicting larger cell height. From our findings, we were unable to conclude which group may indicate cells with high vitality for *A. fusiformis*, which supports the inconsistencies between cell and filament categories. The distinct changes in the prevalence of different cell sizes in L. Nakuru can be seen as a reaction to changes in environmental conditions, which, however, does not mean that cells are exposed to adverse conditions. L. Bogoria, on the other hand is both physically and chemically more stable (Harper et al. 2003). The lake′s large volume enhances buffering capacity to the chemical changes expected to accompany rainfall dilution (Schagerl and Oduor 2008) and therefore *A. fusiformis* cells are not confronted by pronounced changes in the physical and chemical variables of the water column. This is well illustrated by the observed lack of big variations in the proportions of each of the cell categories.

From the PCA biplot, it was observed that only nonbiological factors played a significant role in the cell category pattern along the gradients. Biological factors such as *A. fusiformis*-eating zooplankton and cyanophage infections were considered in the post hoc analysis but were not found to contribute significantly to the cell category pattern. Elevated conductivity, temperature, and SRP concentration promoted the percentage of large and medium cells while wind speed coincided with elevated percentage of large cells. *A. fusiformis* develops aerotops to regulate its position along the underwater light gradient and follow the daily and seasonal light changes (Vonshak and Tomaselli 2000). Although we were not able to obtain data concerning gas vesicle development, we assume that increased cell size results in a higher potential to form gas vesicles and therefore a better buoyancy regulation to counteract turbulent conditions. The importance of advection and turbulence has also been reported for marine ecosystems (Li 2002). The author observed that phytoplankton size structure across various marine ecosystems varies with the ocean physics, which set the nutrient supply and irradiance levels to which phytoplankton are exposed.

Elevated NO_3_-N amounts coincided with small cells, which indicated that NO_3_-N uptake was rather low when small cells prevailed. Such a pattern has also been observed for diatoms in earlier laboratory studies (Stolte and Riegman 1995): smaller diatom species had lower nitrate uptake rates and lower intracellular pools compared to the larger diatom taxa.

### Filament dimensions

The temporal trend of filament morphology groups in L. Nakuru seemed to have been influenced by the frequent changes in physicochemistry and biological parameters. During stressful conditions, *A. fusiformis* filaments were damaged leading to filament breakage as was seen in the increase in small filaments probably making them more vulnerable to, for example, cyanophage attacks (Peduzzi et al., unpublished data). It was during such phases that *A. fusiformis* was out-competed by other phytoplankton groups (*Anabaenopsis*, cryptomonads, and other cyanobacteria) and thus the occurrence of *Arthrospira* crashes. Wu et al. (2005) similarly observed breakage of filaments and photoinhibition when *Arthrospira* filaments were exposed to ultraviolet radiation.

On the other hand, largeW and largeN filaments thrived during *A. fusiformis* peaks depicting favorable conditions for *A. fusiformis* occurrence. A number of studies have demonstrated the adaptive morphological changes from loosened to tightened helix that *A. fusiformis* manifests under various environmental conditions such as, light, temperature, and salinity. For instance, loose or straight *A. fusiformis* forms can change to tight helical forms under high light intensities (Bai and Seshadri 1980) from about 8 h (Gao et al. 2008) to 4–5 days (Helbling et al. 2006). In laboratory experiments, Bai and Seshadri (1980) observed two main forms (S-type corresponding to largeW and H-type identical to largeN), which they could convert via an intermediate C-type. Whereas the intermediate types seemed to be promoted by high nutrient supply, both S- and H-types were increasing during nutrient depletion. It was mainly irradiance, which promoted either loosely coiled (S-type, low light) or densely coiled (H-type, high light) filaments. Wu et al. (2005) suggested that a reduced pitch could be an effective protective mechanism against self-shading to counteract ultraviolet or high photo-active radiation levels. During our field study, irradiance supply per se did not separate largeW and largeN filaments, but wind speed did. Turbulences induced by elevated wind speed seem to be a key variable; the filaments are exposed more in dark areas of the water body and as a result, the pitch increases. During calm periods, cells tend to accumulate near the surface scums due to buoyancy forming densely coiled spirals as a protection against excess radiation. It is not only light penetration, but also the turbulence regime, which controls the shape.

Additionally, zooplankton might influence the pitch: an increase in *A. fusiformis*-eating zooplankton was coinciding with largeN filaments. Grazing is one of the most widely explored environmental constraints on size and shape spectrum of phytoplankton as it plays a key role in the size-scaling of phytoplankton (e.g., Salmaso and Padisák 2007; Stoyneva et al. 2007). Burian et al. (2013) observed that zooplankton ingests *A. fusiformis* filaments in a spaghetti-like way. As densely coiled filaments are harder to grasp, the observed coincidence could be a reaction of *A. fusiformis* to reduce the grazing pressure. Such defense strategies of phytoplankton against grazing by the rotifer *Brachionus* have already be proved for green algae such as *Scenedesmus* (Verschoor et al. 2004) and *Micractinium* (Luo et al. 2005); it is assumed that kairomones released by the rotifers promote defense structures like bristles.

The explanatory value of cyanophages is weak, but still significant (Fig. 6). It is an indicator that cyanophages attacking *Arthrospira* are increased especially during periods of high *A. fusiformis* biomass, which is in accordance to other findings, as virus replication rates usually increase in conjunction with increases in host growth rates (Suttle 2007). In *A. fusiformis*-dominated natural systems, this study therefore is the first to provide evidence of massive cyanophages attacks even though this aspect needs further in-depth studies on the phage–host relationship.

*F*_*v*_/*F*_*m*_ values were within the typical range of cyanoprokaryotes (Campbell et al. 1998), which were with a few exceptions the dominant phytoplankton group throughout the investigation period (Fig. 3). For this group, lower *F*_*v*_/*F*_*m*_ of 0.40 to 0.60 is common, because *F*_0_ increases especially at higher phycocyanin contents (Campbell et al. 1998) thus lowering *F*_*v*_. A comparison between filament type groups and *F*_*v*_/*F*_*m*_ revealed a clear pattern: large filaments, especially largeW, were highly related to increases in *F*_*v*_/*F*_*m*_. As this group is generally dominating the community in Bogoria and sometimes in Nakuru, we conclude that largeW indicates filaments of high vitality; this is also the case for largeN. Contrarily, small filaments are related to lower *F*_*v*_/*F*_*m*_, which can be seen as indicator for adverse conditions.

Summarizing up, the study was able to demonstrate detailed morphological changes of *A. fusiformis* in nature for the first time. Key variables responsible for the morphological changes were identified. More so, from morphology changes, a pronounced shift in *A. fusiformis* biomass can be deduced: largeW and largeN filaments indicating filaments of high vitality prevailed during the periods of *A. fusiformis* peaks.

It was clearly demonstrated that morphological changes reflected the abiotic and biotic developments that took place in the two lakes during this study. Therefore, *A. fusiformis* morphotypes may reliably be used as one of the easily accessible monitoring tools for the prevailing environmental and biological variables in soda lakes as its shape and structure is highly subjective and therefore reflective of what is happening in its habitat. As an additional benefit, our findings may also be of interest for mass cultivation, as morphological features provide insight into the vitality of the cultures and induced changes of the filament shape might be used for optimizing harvesting.
